# Nuclear and Mitochondrial Epigenetic Mechanisms Underlying Neurodegeneration and Gut–Brain Axis Dysregulation Induced by Micro- and Nanoplastics

**DOI:** 10.3390/genes17020151

**Published:** 2026-01-28

**Authors:** Dragica Pavlovic, Dragana Papic, Vladimir Janjic, Marina Mitrovic, Milica Dimitrijevic Stojanovic, Marina Gazdic Jankovic

**Affiliations:** 1 Department of Genetics, Faculty of Medical Sciences, University of Kragujevac, 69 Svetozar Markovic Street, 34000 Kragujevac, Serbia; drmiloradovic7@gmail.com (D.P.); or marinagazdic@medf.kg.ac.rs (M.G.J.); 2 Center for Harm Reduction of Biological and Chemical Hazards, Faculty of Medical Sciences, University of Kragujevac, 69 Svetozar Markovic Street, 34000 Kragujevac, Serbia; 3 Department of Psychiatry, Faculty of Medical Sciences, University of Kragujevac, 34000 Kragujevac, Serbia; vladadok@fmn.kg.ac.rs; 4 Psychiatry Clinic, University Clinical Center Kragujevac, 34000 Kragujevac, Serbia; 5 Department of Medical Biochemistry, Faculty of Medical Science, University of Kragujevac, 34000 Kragujevac, Serbia; marina.mitrovic@fmn.kg.ac.rs; 6 Center for Molecular Medicine and Stem Cell Research, Faculty of Medical Sciences, University of Kragujevac, 34000 Kragujevac, Serbia; milicadimitrijevic@yahoo.com; 7 Department of Pathology, Faculty of Medical Sciences, University of Kragujevac, 34000 Kragujevac, Serbia

**Keywords:** plastics, microplastics, nanoplastics, gut–brain axis, neurotoxicity, epigenetic mechanisms, neurodegeneration

## Abstract

The increasing and global distribution of microplastics and nanoplastics (MPs/NPs) in the environment has led to concern about their potential influence on human health, especially on the gastrointestinal tract, as well as the brain. MPs/NPs could traverse epithelial and endothelial barriers, disrupt the gut microbiota, and perturb the microbiota–gut–brain axis, leading to systemic inflammation and possibly extending neurodegenerative processes. Experimental models now demonstrate that MPs/NPs reprogram nuclear and mitochondrial epigenetics—DNA methylation, histone modifications, non-coding RNAs, and mitochondrial DNA regulation—in gut, immune, and neural cells with downstream effects on synaptic function, neuronal survival, and protein aggregation. This mechanistic narrative review integrates preclinical and emerging human evidence of how MPs/NPs compromise intestinal barrier integrity, modulate gut microbiota composition, affect the blood–brain barrier, and converge on oxidative stress, neuroinflammatory signaling, and cell death pathways within the central nervous system across key neurodegenerative diseases. Overall, the review offers an integrated model in which environmental exposure to chronic MPs/NPs disrupts the microbiota–gut–brain axis and drives concurrent nuclear and mitochondrial epigenetic remodeling, lowering the threshold for neurodegeneration in susceptible individuals, while outlining candidate mechanistic readouts that require exposure-specific validation in human-relevant models and longitudinal cohorts.

## 1. Introduction

By 2050, global plastic manufacturing is expected to reach approximately 25 billion tons of garbage per year [1]. Plastic fragmentation can occur due to physical, chemical, or biological causes, resulting in microplastics (MPs, 1 µm–5 mm) [2] and nanoplastics (NPs, <1 µm) [2,3,4]. Particles 50–100 nm in diameter can enter the intestinal villi, whereas larger particles (300 nm to 3 µm) are taken up by Peyer’s patches. Particles smaller than 10 µm may breach the gut barrier and enter the bloodstream, and have been associated in experimental and limited human data with gut microbiota dysbiosis, immune activation, and tissue distribution, depending on size, surface chemistry, and dose [2]. In addition to consuming contaminated food and drinking water as the primary route of exposure to MP/NPs [5], individuals are also exposed to plastics by inhalation [6] and skin contact [7]. Existing overviews primarily focus on general neurotoxicity, blood–brain barrier (BBB) damage, oxidative stress, and neuroinflammation reported after MPs/NPs, but they treat these domains independently and pay little attention to mitochondrial epigenetic or translational indicators [8,9,10]. Most mechanistic evidence currently derives from animal and in vitro studies using selected polymers and exposure paradigms; therefore, the framework should be interpreted as hypothesis-generating and sensitive to dose/property choices. Selective publication of positive toxicity findings may inflate perceived effect consistency and magnitude; we therefore emphasize evidence-stream grading and cautious causal language throughout. Additionally this narrative mechanistic review focuses on evidence suggesting that MPs/NPs can disrupt gut barrier integrity and microbiota composition, perturb microbiota–gut–brain axis, and are associated with nuclear and mitochondrial epigenetic changes (DNA methylation, histone modifications, non-coding RNAs) in gut, immune, and neural cells—predominantly in preclinical systems. These mechanisms are framed within neurodegenerative diseases. Our goal is to provide an integrated mechanistic framework describing plausible pathways by which MPs/NPs exposure could contribute to microbiota–gut–brain axis disruption and epigenetic remodeling, thereby potentially influencing neurodegeneration-relevant biology, and in that manner outlining candidate mechanistic readouts that require exposure-specific validation in human-relevant models and longitudinal cohorts.

## 2. Materials and Methods

### 2.1. Review Design

This manuscript is a mechanistic narrative review following non-systematic review methods, with quality domains guided by the SANRA evaluation tool. The objective was to develop an integrated mechanistic framework linking micro- and nanoplastics (MPs/NPs) exposure with gut–brain axis perturbations and nuclear/mitochondrial epigenetic dysregulation relevant to neurodegeneration biology, rather than to perform quantitative pooling of effect sizes. Accordingly, no meta-analysis was undertaken.

### 2.2. Literature Search

We searched PubMed/MEDLINE, Web of Science, Scopus, and Google Scholar until 30 November 2025 using free-text terms related to microplastics and nanoplastics, gut and blood–brain barriers, the microbiota–gut–brain axis, neurotoxicity, oxidative stress, mitochondrial dysfunction, and nuclear/mitochondrial epigenetic mechanisms in major neurodegenerative diseases. To improve coverage, we also screened bibliographies of significant original publications and relevant review papers.

### 2.3. Eligibility Criteria

Eligible papers included peer-reviewed experiments in vivo and in vitro, human biomonitoring studies, epidemiological reporting on MPs/NPs in biological matrices, and mechanistic work on redox, mitochondrial, and epigenetic pathways with clear relevance to neurodegeneration. We excluded conference abstracts, non-peer-reviewed publications, and papers without mechanistic or gut–brain relevance.

### 2.4. Study Screening and Selection

Retrieved records were screened at the title/abstract level for relevance to the predefined mechanistic scope of the review, and full texts were assessed when needed to confirm mechanistic and gut–brain/neuro relevance. Screening and study selection were performed by the authors, and any uncertainties were resolved by discussion and consensus. Articles were scanned manually and selected applying a date range from 1995 to 2025. When multiple publications addressed closely related exposure paradigms, priority was given to studies with clearer exposure characterization (e.g., polymer, particle size range, dose, and exposure duration) and mechanistically informative endpoints.

### 2.5. Data Extraction and Qualitative Synthesis

For included studies, we extracted key descriptive information (study type and model/species, tissue/biological system, polymer/particle characteristics when reported, exposure route/dose/duration, and the main mechanistic endpoints and directionality of effects). Extracted information was synthesized qualitatively across mechanistic themes to support pathway-level integration and the construction of figures/tables rather than effect-size estimation.

### 2.6. Evidence-Stream Grading and Reporting Boundaries

Key mechanistic statements were linked where possible, and major claims were graded by level of evidence (human, animal, in vitro, or inferred) to clarify translational distance and guide cautious wording. This grading also informed visual encoding in the conceptual model (solid arrows: relatively well-supported steps in experimental models; dashed arrows: proposed or incompletely resolved directionality; dotted arrows: mechanistic overlap/disease relevance rather than proof of causality). The purpose was to develop an integrated mechanistic framework rather than to exhaustively catalogue all observational studies; hence, no formal study registration, risk-of-bias assessment, PRISMA flow diagram, or quantitative meta-analysis was undertaken.

## 3. Current Human Evidence and Inferred Risk

Up until now, research on micro- and nanoplastics in humans has largely addressed two descriptive questions: in which biological matrices can these particles be detected, and can their internal burden be quantified? MPs/NPs have been reported in several human tissues and fluids, including stool, blood, placenta, lung tissue, and cardiovascular tissues [11,12,13,14,15]. More recently, MPs have been detected in decedent human brains and in the cerebrospinal fluid of living patients [16,17]. Detection in brain tissue showed concentrations 7–30 times higher than in the liver or kidneys, with significantly elevated levels in individuals with dementia compared to controls [16,17]. In a parallel study, four types of MPs—polypropylene, PVC, polyethylene, and polystyrene—were identified in cerebrospinal fluid samples from both AD patients and cognitively normal controls, with polyethylene and PVC levels significantly elevated in AD patients [17]. CSF polyethylene levels correlated inversely with cognitive performance and cerebrospinal fluid Aβ42 concentrations, while higher polyethylene levels predicted faster cognitive decline over one year of follow-up [17].

Collectively, these observations support systemic distribution and possible access of plastic particles to the CNS/CSF and also indicate possible accumulation within neural tissues. Current discussions of potential brain risk are commonly organized around three evidence streams. First, biomonitoring studies report MPs/NPs (or polymeric particles) in matrices consistent with gastrointestinal exposure and systemic distribution, including blood and, more recently, neural tissue and CSF [12,13,14,15,16,17]. Second, observational human studies report associations between MPs in carotid atherosclerotic plaques and inflammatory markers (e.g., IL-6, IL-12p70), as well as an association with subsequent cardiovascular events; e.g., the first evidence emerging that MPs within carotid atherosclerotic plaques are associated with an enhanced risk for myocardial infarction, stroke, and all-cause mortality during follow-up [18,19,20]. In parallel, emerging data link plastic particle exposure and/or internal burden to gastrointestinal barrier dysfunction or inflammation, although causality remains unresolved [2,21]. Third, multiple animal and in vitro studies report gastrointestinal barrier alterations, dysbiosis, oxidative stress and mitochondrial perturbations, BBB-related endpoints (including altered tight-junction protein expression), and epigenetic alterations following exposure to selected polymers, particle sizes, and doses; nevertheless, these findings vary across models and exposure paradigms, and their translational relevance to human neurodegenerative outcomes remains uncertain [10,22,23,24,25,26,27,28,29,30]. Accordingly, the gap between intermediate phenotypes and neurodegenerative endpoints remains a central translational challenge and a priority for the next phase of human studies.

## 4. Impact of Micro/Nanoplastics on Intestinal Epithelial Barrier Dysfunction, Intestinal Immune Dysregulation and Gut Dysbiosis

### 4.1. Intestinal Epithelial Barrier Dysfunction

Plastics can infiltrate the food supply and may include compounds that disrupt intestinal equilibrium. Polyethylene (PE), polypropylene (PP), polystyrene (PS), polyvinyl chloride (PVC), and polyethylene terephthalate (PET) are the most commonly found polymers in food and drinking water, with polyamide, acrylics, various polyesters, and polymethyl methacrylate (PMMA) appearing less frequently [31]. MPs, primarily from the PP and PET groups, are present in human feces at a concentration of 20 microplastic particles per 10 g stool (50–500 µm), indicating daily consumption of contaminated meals [11]. The effects of MPs/NPs on human health at ecologically relevant concentrations are unknown. Thus, it is difficult to assess health risks and kinetic plausibility of proposed MP/NP-induced molecular mechanisms of toxicity from the real world because spectroscopy reports micro/nanoparticles by number, size, shape, and polymer type, while exposure science typically reports doses by mass. Most recently, the summed total daily intake of MPs from drink and the most commonly eaten food (meat, grains, fruit, and vegetables) was estimated to range between 7.7 × 10^−3^ and 3.8 × 10^8^, with the median value of 721 #MPs/kg of bw/day [32]. By transforming microplastic counts into a mass value relevant to human toxicology, Senathirajah et al. (2021) found that globally on average, humans ingest 0.1–5 g of microplastics weekly through various exposure pathways; that is approximately just over 250 g a year [33]. Interestingly, even daily use of plastic cutting boards may result in an estimated per-person annual exposure of approximately 7.4–50.7 g of MPs due to abrasion during food preparation [34]. Because of their chemical stability and absence of digestive enzymes, MPs/NPs degrade minimally during transit. MPs larger than 150 μm adhere to the mucus layer and make contact with the apical surfaces of enterocytes, whereas smaller particles can easily diffuse or penetrate toward the epithelium, depending on the properties of the surface [35]. According to the results from animal and in vitro studies, it has been noted that the uptake of MPs/NPs is size-dependent and involves transcytosis by M cells in Peyer’s patches, endocytosis by enterocytes, persorption by villus tips, and paracellular transit via loosened tight junctions [35,36,37,38]. The majority of particles are removed in feces after intake, although a tiny fraction can remain in the colon for several days. Animal and experimental animal studies support size-dependent translocation and tissue distribution under selected exposure conditions and report gastrointestinal barrier perturbation and inflammatory signaling in some models; however, direct evidence for persistent low-grade inflammation in humans at environmentally relevant exposures remains limited, and reported effects vary across polymers, particle properties, and doses [36,37,38]. Overall, substantial data on barrier disruption come from rodent gut and in vitro intestinal models, with limited direct evidence in humans. As a result, in humans, MPs/NPs are found in feces and have been linked to the detection of gut inflammation and IBD markers, implying that barrier disruption is a possible mechanism, but causality or neurological effects have yet to be confirmed [39]. Most information on intestinal barrier disruption comes from animal and in vitro models employing specified polymers and supra-environmental dosages. However, important gaps remain in exposure quantification, dose relevance, and interspecies variability in gut physiology [21,40,41,42,43,44]. 

### 4.2. Gut Immune Impact

**Figure 1:**
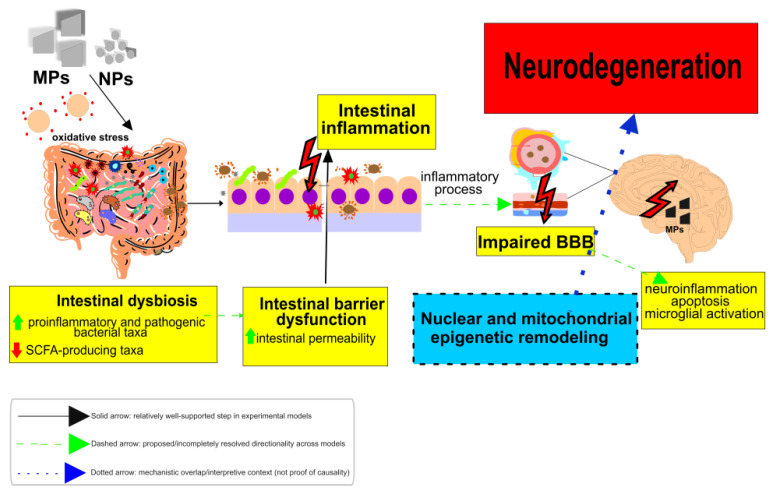
[Animal + In vitro + Inferred] Micro- and nanoplastics (MPs/NPs) may disrupt the microbiota–gut–brain axis. MPs/NPs may promote intestinal oxidative stress, inflammation, barrier dysfunction (increased intestinal permeability), and dysbiosis (often reported as increased proinflammatory/pathogenic bacterial taxa with reduced SCFA-producing taxa), enabling particles, lipopolysaccharide (LPS), and cytokines to enter the circulation and potentially impair blood–brain barrier (BBB) integrity. Converging gut-, immune-, and brain-derived signals may drive nuclear and mitochondrial epigenetic remodeling (DNA methylation, histone modifications, and non-coding RNAs) and, together with oxidative stress and neuroinflammation, promote microglial activation, apoptosis, and neurodegeneration-relevant outcomes. Solid arrows indicate steps relatively well supported in experimental models; dashed arrows indicate proposed or incompletely resolved directionality across models; dotted arrows indicate mechanistic overlap/interpretive context rather than proof of causality. ↑ively well decrease. Abbreviations: BBB, blood–brain barrier; MPs, microplastics; NPs, nanoplastics; SCFAs, short-chain fatty acids.

Lehner et al. (2020) developed a 3D in vitro intestinal model utilizing human intestinal epithelial cell lines (Caco-2) co-cultured with mucus-secreting HT29-MTX-E12 cells to investigate the biological effects of orally relevant MPs exposure under regulated conditions [45]. The findings demonstrated modifications in the concentrations of inflammatory cytokines (IL-8, TNFα, and IL-1β) and barrier integrity; yet, these variations did not cause significant changes [45]. Conversely, in one in vitro study PP-MPs (50–500 µm) were reported to elicit immune responses by stimulating the production of proinflammatory cytokines, including IL-6 and TNF-α (Figure 1) [46]. Research on mouse models indicates that exposure to PE MPs results in alterations in the levels of IL1α and granulocyte colony-stimulating factor (G-CSF) in the bloodstream [47]. Additionally, one study displayed that regulatory T-lymphocyte numbers decreased and Th17 cell proportions increased in the spleen [47]. The same study demonstrated that PE-MPs (10–150 μm) at high doses (600 μg/day) caused inflammation by increasing Toll-like receptor 4 (TLR4), activator protein-1 (AP-1), and Interferon Regulatory Factor 5 (IRF5) expression (Figure 1). When exposed to MPs, serum IL-1α levels increased, and the percentage of Th17 and Treg cells decreased with no significant change in the Th17/Treg ratio [47]. However, it must be noted that the majority of evidence for these immunological alterations is sourced from investigations on murine gastrointestinal systems, where data acquired from intestinal and immune cell lines is highly congruent. In different experimental animal models, MPs and NPs have been found to impair intestinal barrier function and to affect mucosal immune signaling [37,43,44]. Greater pro-inflammatory changes are evident in the case of high doses and/or continued exposure. Disruptions in barrier and immunological function may promote systemic inflammatory signaling, potentially via increased translocation of particles, microbial products, and cytokines. This systemic signaling might be an upstream player in the microglial priming, vascular dysfunction, and neuronal susceptibility of the brain, suggesting that there are early-stage connections between gut exposure to MPs and NPs and subsequent neurodegenerative processes [21,48]. Several in vivo and in vitro studies report activation of NF-κB-linked inflammatory transcription alongside suppression of Nrf2-regulated antioxidant responses in intestinal tissues or intestinal cell models following MPs/NPs exposure, particularly under higher-dose and/or prolonged exposure conditions, although the direction and magnitude of these changes vary across models [49,50,51,52].

**Figure 2:**
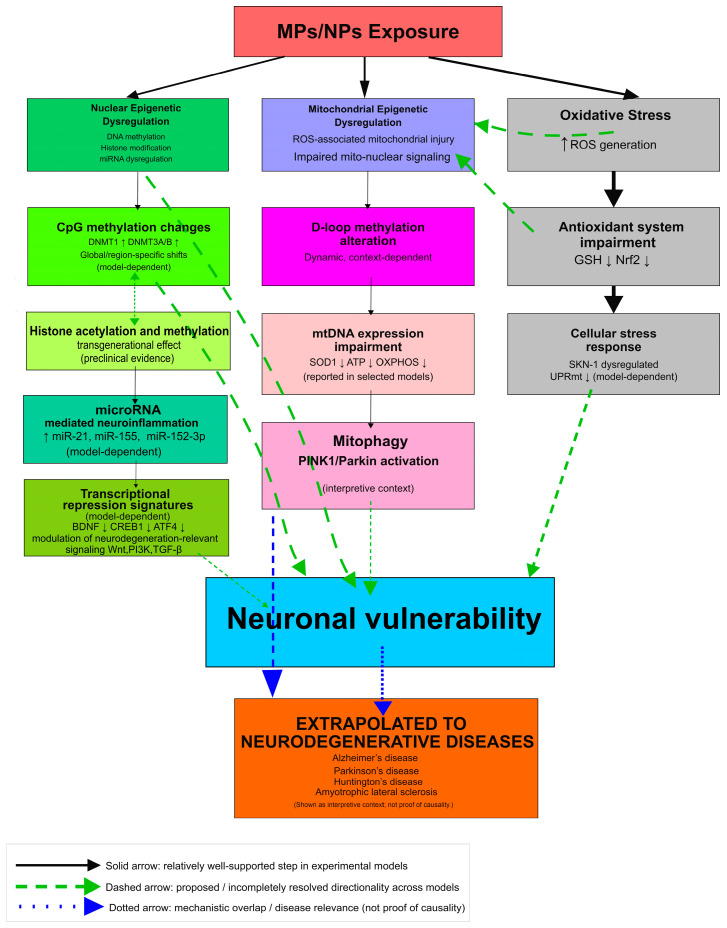
Proposed nuclear-mitochondrial epigenetic framework linking micro- and nanoplastic (MPs/NPs) exposure to neurodegeneration-relevant pathways and increased neuronal vulnerability. MPs/NPs exposure is shown to converge on nuclear epigenetic dysregulation, mitochondrial epigenetic dysregulation (including impaired mito-nuclear signaling), and oxidative stress via increased ROS generation. Within the nuclear arm, CpG methylation changes (DNMT1 ↑, DNMT3A/B ↑; global/region-specific shifts; model-dependent), histone acetylation and methylation (with transgenerational effect indicated as preclinical evidence), microRNA-mediated neuroinflammation (miR-21, miR-155, miR-152-3p; model-dependent), and transcriptional repression signatures (BDNF ↓, CREB1 ↓, ATF4 ↓; model-dependent) are depicted as converging signals. Neurodegeneration-relevant signaling pathways (Wnt, PI3K, TGF-β) are included as interpretive context within the transcriptional module. In the mitochondrial arm, D-loop methylation alteration (dynamic, context-dependent) is linked to mtDNA expression impairment (reported in selected models) and mitophagy, with PINK1/Parkin activation indicated as interpretive context. Solid arrows denote relatively well-supported steps in experimental models, dashed arrows denote proposed or incompletely resolved directionality across models, and dotted arrows indicate mechanistic overlap or disease relevance without implying causality. Neuronal vulnerability is extrapolated to neurodegenerative diseases (Alzheimer’s disease, Parkinson’s disease, Huntington’s disease, and amyotrophic lateral sclerosis) strictly as interpretive context and not as proof of causality. ↑ increase ↓ decrease Abbreviations: MPs/NPs, microplastics/nanoplastics; ROS, reactive oxygen species; CpG, CpG dinucleotides (CpG sites); DNMT, DNA methyltransferase; DNMT1, DNA methyltransferase 1; DNMT3A/B, DNA methyltransferase 3A/3B; miR, microRNA; BDNF, brain-derived neurotrophic factor; CREB1, cAMP response element-binding protein 1; ATF4, activating transcription factor 4; Wnt, Wnt signaling; PI3K, phosphoinositide 3-kinase; TGF-β, transforming growth factor beta; D-loop, displacement loop (mtDNA control region); mtDNA, mitochondrial DNA; SOD1, superoxide dismutase 1; ATP, adenosine triphosphate; OXPHOS, oxidative phosphorylation; PINK1, PTEN-induced kinase 1; Parkin (PRKN), parkin RBR E3 ubiquitin protein ligase; GSH, glutathione; Nrf2, nuclear factor erythroid 2–related factor 2; SKN-1, SKN-1 (*C. elegans* Nrf2 ortholog); UPRmt, mitochondrial unfolded protein response; AD, Alzheimer’s disease; PD, Parkinson’s disease; HD, Huntington’s disease; ALS, amyotrophic lateral sclerosis. See paragraph above for competing hypotheses and falsifiable predictions.

Because Figure 1 and Figure 2 integrate heterogeneous evidence streams, the framework should be interpreted as hypothesis-generating rather than causal. To improve falsifiability, we highlight that the proposed cascade would be weakened if environmentally relevant internal burdens do not track with validated markers of epithelial/BBB dysfunction and systemic inflammation, if CNS nuclear/mitochondrial epigenetic readouts occur without peripheral immune/oxidative activation, if adjustment for additives/co-pollutants abolishes observed associations, or if longitudinal cohorts show no temporal relationship between baseline MPs/NPs burden and neurodegeneration-relevant endpoints. These constraints are intended to limit over-interpretation and clarify which observations would support—or argue against—the proposed mechanisms.

### 4.3. Microbiota Effect

There is limited direct information on how MPs/NPs exposure affects the human gut microbiome [48,49]. Experimental studies in mammalian models nevertheless indicate that micro- and nanoplastic exposure, both acute and longer-term, can perturb gut microbial community composition and diversity, with the extent and course of change depending on polymer type, particle size/chemistry, dose, and exposure duration [49,53]. In murine studies, dysbiosis—broadly defined as a disruption of the normal microbiota balance—is reported frequently and is typically reflected in changes in alpha diversity and in community structure (beta diversity), although the direction and magnitude of these shifts vary across studies [53]. MPs exposure in animal studies was summarized as being associated with shifts in dominant phyla (e.g., Bacteroidetes, Firmicutes, Actinobacteria, Proteobacteria) and with genus-level differences that included Staphylococcus, Clostridium, and Bacteroides, with study-specific changes reported across multiple taxa (Figure 1) [53]. Exposure to MPs and NPs has been associated with gut dysbiosis in animal models where there is less diversity of microbes with lower levels of short-chain fatty acid (SCFA)-producing taxa and higher amounts of likely pro-inflammatory bacteria [53]. On the other hand, available data for humans is scarce and heterogeneous. Collectively, these findings suggest that these microbiota alterations may be reflected in changes in microbial metabolite synthesis and neurotransmitter precursor availability, thereby influencing epithelial integrity, immune response, and brain activity through the gut–brain–microbiota axis [21,22,54,55].

## 5. Microbiota–Gut–Brain Axis Metabolites as Potential Epigenetic Mediators Linking MP/NP Exposure to Neurobiological Outcomes

The gut–brain axis is a multifaceted bidirectional communication network linking the gastrointestinal tract and the central nervous system through microbial, immune, neural, and endocrine routes [56]. The gut microbiota contributes to host tryptophan metabolism and, through its metabolic outputs, can influence neuromodulatory and neurotransmitter-related pathways, including signals relevant to serotonergic and GABAergic systems [57]. In preclinical studies, chronic low-dose exposure to polystyrene nanoparticles has been reported to coincide with neuroinflammatory and apoptotic readouts, alongside behavioral changes interpreted as anxiogenic-like phenotypes in rats [58]; in the same experimental settings, exposure was also associated with shifts in gut microbiota composition. More broadly, available animal and in vitro evidence suggests that MPs/NPs exposure may be accompanied by dysbiosis, altered intestinal barrier function, and increased inflammatory signaling; in some models, these systemic changes co-occur with findings consistent with BBB dysfunction, although directionality and causality remain insufficiently resolved (Figure 1) [59]. A frequently discussed working hypothesis is that peripheral inflammatory mediators and microbe-derived products (e.g., LPS), if translocated into the circulation under conditions of barrier impairment, could contribute to neuroimmune activation and thereby exacerbate neuroinflammatory phenotypes [60]; however, direct, model-consistent evidence demonstrating this entire cascade specifically in the context of MPs/NPs exposure is still limited. Independently of plastic exposure, microbiota-derived metabolites are recognized modulators of epigenetic regulation within the microbiota–gut–brain axis. Short-chain fatty acids (SCFAs)—particularly butyrate and propionate—have been shown, in a context- and concentration-dependent manner, to influence histone acetylation, including via HDAC-related mechanisms, thereby affecting gene regulation in epithelial and immune cells and plausibly shaping neuroimmune communication [61,62,63,64]. In parallel, tryptophan-derived metabolites (e.g., indole derivatives) and bile acids can signal through receptors such as AhR, PXR, and FXR, enabling transcriptional programs relevant to barrier integrity and inflammatory tone [65,66,67,68]. If MPs/NPs exposure produces sustained dysbiosis in a given model, it is biologically plausible that downstream metabolite pools (SCFAs, tryptophan metabolites, bile acids) and stress- or inflammation-responsive pathways (e.g., NF-κB/Nrf2-associated signaling and metabolite-sensitive epigenetic regulation) may shift accordingly. Together, such shifts could plausibly bias transcriptional programs toward a more inflammatory and stress-responsive state; however, whether these changes are sufficient to meaningfully modify neurodegenerative trajectories remains unproven, particularly in humans [38,69,70,71,72,73,74]. Nevertheless, most supporting evidence remains preclinical, and current data are insufficient to infer that these microbiota–metabolite–epigenetic perturbations initiate or accelerate neurodegenerative disease in humans [74]. Consequently, while reducing plastic exposure may be framed as a precautionary consideration, causal evidence that such measures slow the onset or progression of human neurodegenerative disorders remains limited and requires well-designed longitudinal studies.

## 6. Mechanistic Core: Genetic, Epigenetic, and Mitochondrial Pathways Involved in the Neurotoxicity of MPs and NPs

Current data suggests that micro- and nanoplastics may alter nervous-system biology via several overlapping pathways rather than a single linear mechanism, with mechanistic support derived mostly from in vitro and animal models. In preclinical studies, exposure is frequently associated with oxidative stress and inflammatory signaling, as well as changes in neuronal function and glial responses; in some cases, programmed cell-death phenotypes (most commonly apoptosis and less consistently pyroptosis-like features) have also been reported. At the molecular level, these responses usually correspond with changes in gene expression and epigenetic readouts (DNA methylation, histone modification, non-coding RNAs), as well as mitochondrial epigenetics and mitophagy. Given the variability of models, dosages, and polymers, epigenetic and mitochondrial signals should be considered hypothesis-generating mechanistic readouts in experimental systems, pending replication and validation in human-relevant contexts. The next subsections include structural and inflammatory injury as well as epigenetic reprogramming.

### 6.1. Neuronal and Synaptic Injury

An increasing amount of preclinical evidence indicates that micro- and nanoplastics may disrupt neural architecture and synaptic integrity, with the most direct evidence obtained from zebrafish, Caenorhabditis elegans *C. elegans*, and mouse models [40]. Mice exposed to polystyrene microplastics displayed modifications in the prefrontal cortex’s neuronal cytoarchitecture, characterized by a reduction in Nissl bodies, diminished basal dendritic length and complexity, and decreased dendritic spine density, alongside a decrease in BDNF mRNA expression [75]. In zebrafish embryos, exposure to 500 nm polystyrene microplastics (0.1–10 ppm) was associated with altered swimming behavior patterns and multiple neurotoxicity-related endpoints, including increased apoptosis readouts (acridine orange staining), an apoptosis-associated transcriptional profile (p53, caspase-3, and caspase-9 up; bcl-2 down), reduced acetylcholinesterase activity, elevated nitric oxide levels, altered serotonin and dopamine concentrations, and down-regulated BDNF expression [75]. In *C. elegans*, chronic exposure to UV-aged MPs has likewise been reported to produce neurotoxicity linked to disruption of dopamine, glutamate, and serotonin-associated neurotransmission [76]. These organism-level observations are supported by in vitro work in neuronal-like cells, where polystyrene nanoparticles have been reported to induce cytotoxicity and to impair neurite outgrowth, indicating that neuronal morphology can be directly sensitive under certain exposure conditions [77]. Taken together, the available findings indicate that MPs/NPs exposure can coincide with structural remodeling, reduced neurotrophic support (including BDNF-related changes), and apoptosis-associated molecular signatures across multiple models [75,76,77,78,79,80]; however, the contribution of candidate mediators such as nitric oxide should be treated as mechanistically plausible unless it is tested through pathway-level intervention. At the synaptic level, plasticity depends heavily on glutamatergic signaling, including AMPA receptor–dependent mechanisms. N-methyl-D-aspartate (NMDA) receptors and α-amino-3-hydroxy-5-methyl-4-isoxazolepropionic acid (AMPA) glutamate receptors are involved in the molecular mechanisms that mediate synaptic plasticity [81,82]. In addition to the neurotransmitters, synaptophysin (SYN) and CREB are pivotal modulators of synaptic plasticity [83]. Animal studies displayed that SYN expression, serving as a synaptic repair marker, is influenced by MPs/NPs, resulting in atypical synaptic neurotransmitter release, while CREB functions as the principal transcription factor within the cAMP/PKA/CREB signaling pathway [84]. Of note, the impairment of long-term memory is associated with the loss of CREB function [85]. In that line, in a zebrafish model, Yu et al. found that exposure to MPs/NPs impacted this pathway [82]. Finally, it must be clarified that while reviews of the CNS literature consistently cite synaptic dysfunction as a recurrent endpoint of MPs/NPs neurotoxicity [40], specific pathway claims (for example, CREB-axis perturbations) are best reserved for contexts where the relevant targets have been directly quantified in the model under discussion. The subsequent sections associate these modifications with redox-sensitive transcription factors and epigenetic processes that entrench the damage caused by MPs/NPs exposure.

### 6.2. Oxidative Stress and Neuroinflammation

Multiple studies have found that prolonged contact with MPs/NPs has been associated with increased ROS levels and caused nervous system dysfunction due to factors such as the large surface area of MPs/NPs and the release of pro-inflammatory mediators by additives [40]. Beyond that, in animal studies, investigators reported elevated LPO levels and altered LDH activity in affected brain tissues [86,87]. The disparity between the production rate of LPO and their clearance is generally interpreted as consistent with gradual membrane damage and potential cell death, depending on the experimental context [88]. Research on zebrafish exposed to MPs/NPs has demonstrated a reduction in glutathione (GSH) levels and an elevation in malondialdehyde (MDA) levels, a pattern consistent with LPO [75]. Other studies have linked decreased GSH availability to reduced neuronal survival and less protection against tau protein aggregation; in MPs/NPs exposure settings, this relationship is best framed as biologically plausible rather than universally established [89,90]. According to data from animal studies, MPs/NPs exposure has been reported together with oxidative stress and neuroinflammatory signaling and with shifts in gene-expression profiles; these findings are commonly discussed in relation to redox-sensitive regulators such as Nrf2 and NF-κB and, more cautiously, to epigenetic mechanisms, where HDAC-related pathways are considered plausible modulators rather than consistently demonstrated endpoints [91,92]. Besides the role of antioxidant enzymes, research has displayed that the Nrf2-related pathway is widely regarded as neuroprotective by limiting oxidative stress responses. In zebrafish, fish, and rodent models, exposure to MPs/NPs increased ROS and LPO levels while downregulating Nrf2 pathway targets (e.g., HO-1, GCLC, NQO-1, GCLM), suggesting diminished antioxidant capacity and heightened vulnerability of neurons to oxidative damage [91]. Previous studies have shown MPs/NPs-induced alterations in tight junction proteins (TJPs) and the cellular cytoskeleton, consistent with BBB impairment and, in some animal models, increased brain accumulation/infiltration [25]. TJPs are essential for preserving BBB structure, whereas the cellular cytoskeleton supports its normal architecture [93]. Accordingly, PS-MPs (L-PS group:1 mg/L, M-PS group:10 mg/L, H-PS group: 100 mg/L in water) were assessed to brain tissue damage in chicken after six weeks of continuous exposure [94]. Yin et al. found that activated microglia and astrocytes can stimulate tumor necrosis factor (TNF-α) production in neurons, accompanied by cytoskeletal modifications and reduced BBB integrity [94]. A recent study by Chen et al. in fish also reported increased expression of the matrix metalloproteinase gene MP9, which has been linked to TJP degradation in barrier biology, together with reduced expression of occludin, eNOS, ZO-1, and other TJPs, consistent with weakened BBB integrity (Table 1). Furthermore, the reduction in eNOS levels, which facilitates NO release, may contribute to heightened permeability [87]. Cytokines can rapidly modulate the function of excitatory and inhibitory neurons [95]. Jing’s work demonstrated that exposure to MPs/NPs led to an increase in pro-inflammatory cytokines interleukin-1beta (IL-1β) and TNF-α in Coturnix japonica, and these changes were discussed in relation to excitatory signaling, including NMDA and AMPA receptor subunits [96]. An examination of mononuclear transcription in the brain indicated that the expression of the anti-inflammatory cytokine IL-4 exhibited a dose-dependent decline in mice, consistent with reduced anti-inflammatory signaling in those conditions (Table 1) [97]. IFN-γ, mostly produced by meningeal T lymphocytes, disrupts neuronal transmission [98], while immune cell factors have been demonstrated to significantly influence synapse structure. Notably, C-X3-C motif chemokine receptor 1 (CX3CR1) is a G protein-coupled chemokine identified in microglia. Yang and Liang’s investigation on MPs/NPs in mice found a significant rise in Cx3cr1 gene expression, which was discussed in relation to altered synaptic pruning as well as impaired synaptic stability and maturity [97,99]. In research by Liang et al., IL-4, IL-10, IL-13, IL-1β, and IL-6 exhibited region-specific expression, demonstrating a dose-dependent decrease in the midbrain [97]. So far, the oxidative and inflammatory brain responses to MPs/NPs are mainly documented in zebrafish and rodent brain studies, while data concerning human responses remain insufficient. When sustained, MPs/NPs-driven oxidative stress and neuroinflammation may converge on regulated cell-death programs, including apoptosis and ferroptosis, which are summarized in Section 6.3.

### 6.3. Cell Death Signaling

Across experimental systems, MPs/NPs exposure has been reported together with changes in apoptosis-associated signaling, including components commonly discussed within Fas/FasL-related cascades. In the canonical extrinsic apoptosis pathway, Fas–FasL engagement can promote recruitment of FADD and subsequent activation of caspase-8, with downstream signaling that may converge on execution-phase caspases and apoptotic phenotypes. MPs/NPs exposure has also been linked to mitochondrial apoptosis-related signaling (e.g., cytochrome c–associated pathways) in fish model [87].

Yin et al. noted an elevation in mRNA expression levels of neuronal cell mitochondrial cytc and protein expression levels of Bax, Casp-8, and Casp-3 in chickens subjected to chronic exposure to MPs/NPs [106]. MPs/NPs can also modulate neuronal apoptosis in a p53-dependent manner. Suman et al. and Santos et al. reported a notable elevation in p53 gene transcription levels and a reduction in Bcl-2 gene expression levels in zebrafish neural cells subjected to PS-MPs [75,86]. By controlling the BCL-2 protein, the p53 gene may trigger apoptosis via a cysteine protease-triggered signaling pathway [75]. Conversely, data from in vitro study displayed that the modifications in the activity of the p53 gene might impair the cell cycle checkpoint and modulate DNA damage repair, potentially resulting in neuronal death [107]. Yin et al. proposed that the molecular mechanism by which MPs/NPs induce neuronal pyroptosis entails the marked activation of apoptosis-associated speck-like protein containing a caspase recruitment domain (ASC), nucleotide-binding oligomerization domain-like receptor family pyrin domain protein 3 (NLRP3), and the cleavage of caspase 1 and Gasdermin D (GSDMD), ultimately leading to a substantial increase in the production of IL-18 and IL-1β in chickens [94]. The processes of pyroptosis have been clarified in chickens, while apoptotic mechanisms have been documented in rodent tissues and cell cultures. In contrast, ferroptosis is chiefly deduced from extensive research on nanoparticles and neurodegenerative diseases, rather than being directly evidenced for MPs/NPs in the brain. The transcriptional and epigenetic landscapes that regulate these death pathways have been increasingly acknowledged as dynamic targets of MPs/NPs exposure, as elaborated in Section 6.4.

### 6.4. Epigenetic and Transcriptomic Reprogramming in Neurodegeneration-Relevant Pathways

In gene expression and nanoplastic (NP) exposure experiments [108], Liang et al. provided transcriptomics data from isolated nuclei of frozen brains of adult male C57BL/6 J mice, a typical neurobiology model, after exposure to 50 nm pure PS-NPs [97]. Differentially expressed genes (DEGs) contained Parkinson’s disease-associated genes. DEGs were highest in astrocytes (597 genes), followed by oligodendrocytes (486 DEGs), neurons (326 DEGs), and endotheliocytes (280 DEGs) [97]. The cell-specific inhibition of mitochondrial function, proteostasis, ATP metabolism, and synaptic function was demonstrated with PS-NPs. ATP metabolism was most enriched in neurons, while mitochondrial and proteostasis functions were enriched in oligodendrocytes, astrocytes, and endotheliocytes. Synaptic function modulation was also discovered in astrocytes and endothelial cells. Using cell-specific mechanisms, these authors found that PS-NPs cause PD-like neurodegeneration in mice. In the same animal paradigm, NPs feeding altered gut–brain circadian rhythm gene expression, causing neurotoxicity [84]. Key genes implicated in the neurotoxicity of PS-NPs include *Camk2g*, which encodes a calcium/calmodulin-dependent protein; *Adcyap1*, which encodes essential mediators of neuroendocrine stress responses; and *Per1*, a crucial gene involved in circadian rhythms [84].

Recent research has analyzed gene expression profiles after exposure to NPs utilizing in vitro neuronal cell models. A study identified various metabolic pathways in human neural stem cells (hNS1) subjected to 0.5, 2.5, and 10 μg/mL of PS-NPs over a duration of 4 days. The research identified changes in stress response genes (*hsp27/hspB1*, *hsp70/hspA5*, and *hsp90α*), highlighting a significant rise in mRNA expression of *hsp27/hspB1*, which is essential in AD and MS [109]. Furthermore, 30 nm PS-NPs resulted in modified expression levels of Cu/ZnSOD1 and catalase, as well as inflammatory and mitochondrial responses in the corresponding in vitro neural model [109]. In a separate study, 25 nm PS-NP beads were applied for 24 h to a modified human neuronal model (the SH-SY5Y cell line) engineered to overexpress α-synuclein, a protein implicated in Lewy body formation in PD [110]. The infiltration of PS-NPs into cells further enhanced α-synuclein aggregation, indicating possible implications for human neurodegeneration [110]. The transcriptomic analysis of differentially expressed genes in this three-dimensional in vitro model exposed to 100 nm polystyrene NPs reveals that the Wnt, PI3K-Akt, and TGF-beta signaling pathways are the most enriched [110]. The Wnt and PI3K-Akt signaling pathways are acknowledged in the context of AD [111,112] while TGF-beta is a prevalent characteristic of neurodegenerative disorders, particularly in ALS [113].

Recent studies increasingly point to epigenetic regulation as a plausible link between MPs/NPs exposure and neurodegenerative risk, including AD, PD, and Huntington’s disease [114,115]. In neuronal model systems, exposure has been associated with atypical DNA methylation patterns, altered histone marks, and shifts in microRNA expression- changes that fit with neuronal dysfunction and activation of cellular stress responses (Figure 2). If these changes persist, they further weaken neuronal resilience and help maintain oxidative stress and neuroinflammatory signaling, which are well-recognized contributors to disease progression [116]. Developmental windows are particularly sensitive. A recent study found that even a single 72-h exposure to PS-NPs in *C. elegans* was enough to markedly alter the expression of key germline genes, including *met-2*, *set-2*, and *spr-5*, leading to epigenetic reprogramming and transgenerational effects [117]. In this model, *met-2* and *set-2* were upregulated, while *spr-5* was down regulated. MET-2, the ortholog of mammalian SETDB1, is responsible for regulating H3K9 methylation, whereas SET-2, an ortholog of SET1A/SET1B, governs H3K4 methylation. SPR-5, an ortholog of LSD1/KDM1, mediates the demethylation of H3K4 [118,119,120]. These epigenetic adjustments are important for maintaining proper epigenetic control, which can be disturbed by environmental stressors such as NPs exposure. Liu et al. (2021) showed in *C. elegans* that exposure to PS-NPs increased the expression of CBP-1, which encodes a histone acetyltransferase [121], suggesting that CBP-1-mediated histone acetylation may act as an early epigenetic defense mechanism against NPs-induced stress [121]. CBP-1 may preserve neural stability against environmental insults by modulating critical signaling pathways, including insulin, p38 MAPK, DAF-7/TGF-β, and JNK/MAPK, which govern developmental processes and stress responses [121].

DNA methylation is one of the main epigenetic “switches” that cells use to regulate gene expression. In the brain, it is tightly involved in processes such as synaptic plasticity, memory-related gene programs, and neuronal survival [122]. What has become increasingly clear is that environmental exposures can disturb these patterns, and MPs/NPs are now being discussed in this context (Figure 2). A useful example comes from the fathead minnow (*Pimephales promelas*) [123]. Wade et al. (2025) reported that parental exposure to MPs was followed by altered DNA methylation profiles in F1 offspring that were not directly exposed [123]. The signal was more evident in males and mapped largely to genes tied to basic metabolism, including ribosomal genes and transfer RNAs. If similar changes occur in neural tissues, they could shape neurodevelopmental trajectories and leave the nervous system more vulnerable to later disease [123]. At the mechanistic level, MPs/NPs exposure has also been associated with disrupted CpG methylation in the nuclear genome, often alongside changes in DNA methyltransferase expression or function, particularly DNMT1 and DNMT3A/B (Figure 2). While such epigenetic perturbations are well documented for several classes of engineered nanoparticles, whether the same mechanisms dominate for MPs/NPs in mammalian brain remains under study [124].

MicroRNAs are acknowledged as fundamental regulators of differentiation, activation, and polarization of microglia and macrophages in both normal and pathological conditions of the central nervous system. Inflammatory signals and oxidative stress induced by MPs/NPs exposure are likely the primary factors that modify miR-152–3p levels, thereby initiating this region-specific pathogenesis [116,125]. Dysregulated expression of miR-155 and miR-21 two microRNAs tightly linked to immune and inflammatory signaling-has been associated with heightened neuroinflammation and with processes implicated in the progression of neurodegenerative disorders [126]. These miRNAs are upregulated in PTP cells exposed to PS-NPs, as reported by Barguilla et al. [26]. Downregulation of miR-21 in animal models demonstrates a neuroprotective effect through the reduction in the inflammatory response. miR-155 is frequently reported as upregulated across several neurodegenerative disorders and is widely regarded as a pro-inflammatory miRNA that can amplify neuroinflammatory signaling, with the potential to aggravate disease-associated pathology through inflammation-linked pathways. The roles of miR-21, miR-155, and related miRNAs are well characterized in neurodegeneration and in experimental models of these conditions; however, evidence connecting these miRNAs specifically to micro- and nanoplastics exposure remains comparatively limited and is derived predominantly from rodent studies, in vitro systems, and indirect mechanistic inference rather than from robust human data [127].

### 6.5. Mitochondrial Epigenetic Dysregulation Triggered by Plastics Exposure

Recent studies demonstrated the sensitivity of mitochondrial epigenetic regulation to MPs/NPs exposure [10,28,30]. Mitochondria have vital functions beyond cellular energy production, and they can influence gene regulation via epigenetic or epigenetic-like mechanisms such as mtDNA methylation, mitochondrial non-coding RNAs, and bidirectional communication with the nucleus. These regulatory pathways may exhibit heightened responsiveness in the context of oxidative stress and membrane degradation, as evidenced by observations following MPs/NPs exposure (Figure 2). Unlike nuclear DNA, mtDNA is not protected by histones, potentially making it more vulnerable to environmental assaults. In this context, exposure to MPs/NPs has been investigated as a potential contributor to mtDNA instability and modified methylation in critical mitochondrial regions, such as the D-loop, which regulates mitochondrial gene replication and transcription [28,30,128]. Such changes may be accompanied by poor mitochondrial function and, in neural contexts, reduced cellular stress tolerance; however, these associations are model- and exposure-dependent. Beyond epigenetic effects, MPs/NPs exposure has been connected to mitochondrial ultrastructural and functional damage, such as cristae disruption and membrane potential loss, as well as changes in gene expression associated with mitophagy and mitochondrial biogenesis [108]. These mitochondrial quality-control processes are inextricably linked to Nrf2-dependent redox signaling (Section 6.2) and can be viewed as part of a larger cellular stress-response network rather than a single linear pathway. In mouse models, Xu et al. found that PS-NPs elevated PINK1 and Parkin expression, indicating that mitophagy is activated in response to mitochondrial stress [129]. Under physiological settings, PINK1 accumulates on depolarized mitochondrial membranes and attracts Parkin, resulting in the selective elimination of damaged mitochondria. Chronic overstimulation of this pathway is commonly viewed as a marker of persistent mitochondrial stress and has been linked to dopaminergic neuronal vulnerability in PD models [130]; however, in plastics exposure paradigms, this change is best framed as a mechanistic context rather than an inevitable outcome. Increasing evidence suggests that MPs/NPs can coexist with broader epigenetic changes, such as DNA methylation changes, histone modifications, and non-coding RNA perturbations, in addition to mitochondrial dysfunction [10,28,30]. When taken together, these data support the hypothesis that MPs/NPs may interfere with mito-nuclear communication networks that contribute to neuronal stress resistance [10,30]; however, available evidence does not yet define a single dominating mechanism across models. Plastic NPs can damage *C. elegans* defenses at environmentally relevant quantities (≥1 μg/L). According to Qu et al., PS-NPs (PS-NH_2_ and PS-COOH) caused reproductive damage that persisted across generations [131]. The study also found signs of mitochondrial damage and decreased mitochondrial unfolded protein response (UPRmt). In their dataset, SKN-1 initially reduced stress in parental organisms, but this protective impact diminished with continuous exposure, and the trait became more apparent in offspring [131]. This finding may be related to neurotoxicity since SKN-1 is expressed in dopaminergic neurons. Moreover, reduced SKN-1 signaling in these neurons has been linked to decreased stress tolerance and increased vulnerability to neurotoxic damage, suggesting its role as a stress-resilience node in particle-exposure models [132,133]. A similar result was observed in a fish mixture-toxicity model, where co-exposure to MPs and bisphenol A decreased NRF2-driven antioxidant signaling and lowered SOD1 expression [134]. SOD1 is a significant ALS-associated gene, and carriers of SOD1 mutations have been shown to have altered mitochondrial characteristics (including D-loop methylation) [135] (Table 1 and Table 2). At the same time, these similarities should be viewed as convergence on shared oxidative stress and mitochondrial biology, rather than a direct link between mixture exposure pathways and ALS pathogenesis. The available evidence does not support a universal staging rule in which D-loop hypermethylation consistently reflects “early” mitochondrial dysfunction and hypomethylation reflects “advanced” damage; rather, D-loop methylation appears dynamic and context dependent (e.g., varying between prodromal and later AD stages). Research data have found links between altered D-loop methylation and neurodegenerative disease settings, including AD, while the exact functional and causative role is yet unknown [135]. These findings suggest that mtDNA methylation, particularly in regulatory areas such as the D-loop, may play a role in mitochondrial homeostasis and is frequently described as altered in neurodegeneration.

## 7. Conclusions

Preclinical research suggests that MPs/NPs exposure can alter the epigenetic topography of nuclear and mitochondrial DNA modifications, including methylation, histone marks, and non-coding RNA profiles, in gastrointestinal, immune, and neural tissues. It also affects oxidative stress response genes, synaptic function, mitochondrial quality control, and protein aggregation (animal/in vitro). Molecular alterations occur with changes in gut permeability, microbiota–gut–brain axis signaling, and gut microbiota composition. They may serve as biologically plausible mechanistic pathways, albeit hypothetical, by which prolonged exposure to MPs and NPs could lower the threshold for neurodegenerative and neurodevelopmental phenotypes. However, significant unresolved questions remain about the specificity of these epigenetic markers for MPs and NPs, as well as their reversibility and participation in human neurodegenerative diseases. Longitudinal studies in humans are needed to determine the causal relationship between exposure to MPs/NPs and clinical aspects of AD, PD, and ALS. Finally, studies should focus on standardized outcomes and harmonized exposure metrics, rather than just exposure.

## 8. Limitations

These limitations directly constrain interpretation of our mechanistic framework—particularly exposure relevance/internal dose comparability, causal inference from predominantly preclinical endpoints, and translation of epigenetic/transcriptomic readouts to human neurodegenerative risk—so the proposed model should be considered hypothesis-generating rather than causal. Additionally, selective publication of positive toxicity findings may inflate perceived effect consistency and magnitude, further reinforcing the need for cautious interpretation of the available literature. The majority of mechanistic research has been acquired from non-mammalian and rodent models, which have differing gut microbiomes, BBB, and metabolic processes compared to humans; thus, their translational value is unknown. Humans are exposed to MPs and NPs, which are found in almost all tissues. Despite emerging but limited epidemiological data linking MPs to brain injury in humans, there is an increasing body of neurotoxicological research addressing the epigenetic mechanisms involved. Experimental work involves varying polymers, particle sizes, and doses. However, current tissue analysis procedures are poor, preventing comparisons of MPs and NPs-associated exposure and body burden estimates across studies. The molecular pathways that underpin these effects, such as oxidative stress, inflammation, and nuclear and mitochondrial epigenetic regulatory mechanisms, are poorly understood in mammalian systems, as are the long-term consequences of such perturbations. Furthermore, exposure to many pathways at the same time, as well as co-exposure to plastic additives and adsorbed pollutants, makes determining the precise effects of MPs and NPs challenging. On top of that, the significant latency associated with diseases complicates epidemiological analysis.

## 9. Future Directions

To fully comprehend the impact of MPs/NPs on neurodegenerative diseases, it is critical to better characterize real-world human MPs/NPs exposure (size distributions, polymers, and co-contaminants in air, water, food, and tissues) and embed it in longitudinal cohorts with standardized neurological and cognitive outcomes. Environmentally relevant doses must be used in mechanistic studies that integrate multi-omics and adverse outcome pathway approaches, linking oxidative stress, mitochondrial dysfunction, and epigenetic markers to cellular, neural, and behavioral outcomes in advanced mammalian and human iPSC/brain organoid models. Moreover, it is of utmost importance to systematically map MPs/NPs’ responsive epigenetic changes (DNA methylation, histone marks, and non-coding RNAs) in gut, immune, and neural tissues. It is crucial to standardize sampling, detection, and reporting methods for MPs/NPs and develop less invasive biomarkers (e.g., circulating inflammatory markers, exposure-linked microbiome signatures) to enable large-scale epidemiology across diverse populations and life stages, including transgenerational cohort designs. Finally, to avoid the negative impact of plastics on the brain, it is necessary to investigate preventive and therapeutic strategies along the gut–brain axis (microbiota-targeted interventions, antioxidant and epigenetic modulating diets or drugs, and Nrf2/oxidative stress modulators) and compare polymers, sizes, and surface chemistries, as well as interactions with metals and persistent pollutants.

## Figures and Tables

**Table 1:** Core injury mechanisms of MPs/NPs neurotoxicity and their relevance to neurodegenerative disease pathways.

Mechanistic Axis	Key MPs/NPs-Induced Changes (Models, Endpoints)	Translational Interest Genes/Pathways	Relevance to AD/PD/ALS Epigenetics	Evidence Source
**Oxidative stress and redox signaling**	↑ ROS, lipid peroxidation and NO; ↓ antioxidant enzymes (SOD, CAT, GSH-Px); imbalance between Nrf2-driven antioxidant defenses and NF-κB-dependent inflammatory signaling in brain and gut	Nrf2	↓ Nrf2 activity or mislocalized in AD and PD brains; Nrf2 deficiency worsens amyloid, tau and α-syn pathology in transgenic NDD models	Shown in MPs/NPs models (Animal); well-established Nrf2 dysfunction in AD/PD brains [40,75,86,87,91,92,100,101,102,103].
**Neuronal structure and synapses**	loss of dopaminergic, glutamatergic and GABAergic neurons in zebrafish and rodents; ↓ synaptophysin and CREB expression; cognitive/behaviora deficits	CREB, BDNF	DNA methylation and histone modifications at CREB/BDNF promoters altered in AD/PD/ALS cohorts; changes correlate with synaptic failure and cognitive decline	Synaptic toxicity shown in MPs/NPs models (Animal); CREB/BDNF epigenetic alterations in NDD established [75,77,80,85,91,100,101,104,105].
**Neuroinflammation and BBB dysfunction**	PS-NPs cross or disrupt the BBB in rodent models, activate microglia and astrocytes, upregulate NF-κB, TLR4, and cytokines (IL-1β, IL-6, TNF-α) in CNS and periphery	NF-κB, NLRP3	Chronic NF-κB and NLRP3 inflammasome activation shape disease-associated microglia phenotypes in AD/PD/ALS; these inflammatory programs interact with impaired Nrf2 and dysregulated miR-155	BBB disruption and cytokine induction shown in MPs/NPs models (Animal); microglial NF-κB/NLRP3 and their integration with Nrf2/miR-155 well established in NDD literature [25,87,96,97,98,99,101,104].
**Programmed cell death (apoptosis, pyroptosis)**	activates caspase-3/9, alters BAX/BCL-2 ratio, and triggers apoptosis in neural and non-neural cells; NLRP3-caspase-1-mediated pyroptosis reported in rodent tissues	Caspase-3, NLRP3	Apoptosis, pyroptosis, and emerging ferroptosis contribute to selective neuron and oligodendrocyte loss in AD/PD/ALS models	Apoptosis and NLRP3 activation shown in MPs/NPs models; ferroptosis mechanisms inferred from broader NPs and NDD literature (Animal/in vitro); not yet directly demonstrated in MPs/NPs brain exposure [75,76,86,94,97,106].

AD Alzheimer’s disease; ALS amyotrophic lateral sclerosis; BBB blood–brain barrier; BDNF brain-derived neurotrophic factor; CREB cAMP response element-binding protein; GABA γ-aminobutyric acid; GSH reduced glutathione MPs microplastics; NDDs neurodegenerative diseases; NF-κB nuclear factor kappa-light-chain-enhancer of activated B cells; Nrf2 nuclear factor erythroid 2–related factor 2; ROS reactive oxygen speciesTLR4 Toll-like receptor 4; TNF-α tumor necrosis factor-alpha. ↑ increase, ↓ decrease.

**Table 2:** Key epigenetic and mitochondrial axes linking MPs/NPs exposure to neurodegenerative pathways and their disease-relevance context and testable mechanistic axes.

Axis/Marker	Mechanistic Focus	Evidence for MPs/NPs	Evidence in Human NDDs (AD/PD/ALS)	Potential Measurement Matrices (Examples)
**Nrf2/NF-κB–HDAC–miR-155 axis**	Redox-sensitive inflammatory hub integrating oxidative stress (Nrf2), pro-inflammatory signaling (NF-κB), chromatin remodeling (HDACs), and microglial activation (miR-155).	↑ ROS and NF-κB activation and ↓ Nrf2 target expression in gut, liver, and some brain models; HDAC and miR-155 changes are partly inferred from nanoparticle and gut–brain studies (Inferred); [136].	↓ Nrf2 activity and persistent NF-κB signaling reported in AD and PD brains; miR-155 upregulated in microglia-rich regions and linked to chronic neuroinflammation. [137,138].	Blood cells (Nrf2/NF-κB targets, HDACs), CSF cells; circulating miR-155 in plasma/serum and CSF; exploratory stool extracellular vesicles (EVs) [40].
**DNMT1/DNMT3A/DNMT3B and locus-specific methylation (SOD1, PINK1/Parkin, BDNF/CREB1)**	DNA methylation control of oxidative stress, mitophagy, and synaptic plasticity genes that shape vulnerability to neuronal injury [138].	alter global and gene-specific methylation in developing brain and peripheral tissues; direct MPs/NPs data for SOD1, PINK1/Parkin, BDNF/CREB1 are limited and often inferred from non-MP particle studies (Inferred); [40].	Aberrant global and locus-specific methylation reported in AD, PD, and ALS; altered methylation at SOD1, BDNF, and CREB1 associated with disease risk or progression in some cohorts [139,140].	Blood DNA (global and targeted methylation), post-mortem brain; exploratory host DNA in stool; CSF DNA when available [141].
**mtDNA D-loop methylation and mitochondrial non-coding RNAs (mt-ncRNAs)**	Epigenetic regulation of mitochondrial transcription, biogenesis, and stress responses via D-loop methylation and mt-ncRNAs [138].	mitochondrial swelling, loss of membrane potential, and ROS generation in neural and non-neural tissues; specific changes in D-loop methylation and mt-ncRNAs are mostly extrapolated from broader mitochondrial toxicology and NDD literature (Inferred); [40].	Altered D-loop methylation and dysregulated mt-ncRNAs reported in AD, PD, and ALS brain and blood, linked to impaired mitochondrial function and clinical progression (emerging but growing evidence) [137,138].	Blood (mtDNA methylation, mt-ncRNAs), CSF, post-mortem brain; exploratory measurements in stool (shed cells, EVs) [141].
**miR-21 and miR-155**	Stress- and inflammation-responsive miRNAs that modulate apoptosis, glial activation, and neuron–glia cross-talk [142].	modulate miR-21 and miR-155 expression in several rodent and cell models, mainly in peripheral tissues and developing brain (in vivo + in vitro, limited CNS-focused data) [38].	miR-21 and miR-155 are frequently reported as dysregulated in blood, CSF, and brain of patients with AD, PD, and ALS, and associate with inflammatory activity and clinical status [140,142].	Plasma/serum and CSF (cell-free or EV-associated miRNAs); potentially stool EVs in gut-focused studies [141].

AD Alzheimer’s disease; ALS amyotrophic lateral sclerosis; BDNF brain-derived neurotrophic factor; CNS central nervous system; CREB1 cyclic AMP response element-binding protein 1; CSF cerebrospinal fluid; DNMT DNA methyltransferase; HDAC histone deacetylase; miR microRNA; MPs microplastics; mt-ncRNA mitochondrial non-coding RNA; mtDNA mitochondrial DNA; NDDs neurodegenerative diseases; NF-κB nuclear factor kappa-light-chain-enhancer of activated B cells; NPs nanoplastics; Nrf2 nuclear factor erythroid 2-related factor 2; PINK1 PTEN-induced kinase 1; PD Parkinson’s disease; ROS reactive oxygen species; SOD1 superoxide dismutase 1; ↑ increase ↓ decrease.

## Data Availability

All data are contained within the article.

## Abbreviations

Aβ42, amyloid beta 42; AD, Alzheimer’s disease; AhR, aryl hydrocarbon receptor; ALS, amyotrophic lateral sclerosis; AMPA, α-amino-3-hydroxy-5-methyl-4-isoxazolepropionic acid (receptor); AP-1, activator protein 1; ASC, apoptosis-associated speck-like protein containing a caspase recruitment domain; BBB, blood–brain barrier; BAX, Bcl-2-associated X protein; BCL-2, B-cell lymphoma 2; BDNF, brain-derived neurotrophic factor; CAT, catalase; CNS, central nervous system; CREB, cyclic adenosine monophosphate (cAMP) response element-binding protein; CREB1, cAMP response element-binding protein 1; CSF, cerebrospinal fluid; Cyt C, cytochrome c; CytC, cytochrome c; DNMT, DNA methyltransferase; DNMT1, DNA methyltransferase 1; DNMT3A, DNA methyltransferase 3A; DNMT3B, DNA methyltransferase 3B; eNOS, endothelial nitric oxide synthase; EVs, extracellular vesicles; FADD, Fas-associated death domain; FXR, farnesoid X receptor; GABA, γ-aminobutyric acid; G-CSF, granulocyte colony-stimulating factor; GPR41/43, G-protein-coupled receptor 41/43; GPR109A, G-protein-coupled receptor 109A; GSDMD, gasdermin D; GSH, reduced glutathione; GSH-Px, glutathione peroxidase; HDAC, histone deacetylase; HT29-MTX-E12, mucus-producing human intestinal epithelial cell line (HT29-MTX-E12); IFN-γ, interferon gamma; IL, interleukin; IL-1, interleukin 1; IL-1β, interleukin 1 beta; IL-4, interleukin 4; IL-6, interleukin 6; IL-8, interleukin 8; IL-10, interleukin 10; IL-12p70, interleukin 12p70; IL-13, interleukin 13; IL-18, interleukin 18; IRF5, interferon regulatory factor 5; Keap1, Kelch-like ECH-associated protein 1; LDH, lactate dehydrogenase; LPO, lipid peroxidation; LPS, lipopolysaccharide; MDA, malondialdehyde; miR, microRNA; miR-21, microRNA-21; miR-155, microRNA-155; MMP9, matrix metalloproteinase 9; MPNPs, micro- and nanoplastics; MPs, microplastics; mtDNA, mitochondrial DNA; mtncRNA, mitochondrial non-coding RNA; NDDs, neurodegenerative diseases; NF-κB, nuclear factor kappa-light-chain-enhancer of activated B cells; NMDA, N-methyl-D-aspartate (receptor); NO, nitric oxide; NPs, nanoplastics; NLRP3, NLR family pyrin domain containing 3; Nqo1, NAD(P)H quinone dehydrogenase 1; Nrf2, nuclear factor erythroid 2-related factor 2; PD, Parkinson’s disease; PE, polyethylene; PET, polyethylene terephthalate; PINK1, PTEN-induced kinase 1; PKA, protein kinase A; PMMA, polymethyl methacrylate; PP, polypropylene; PRISMA, Preferred Reporting Items for Systematic Reviews and Meta-Analyses; PS, polystyrene; PXR, pregnane X receptor; ROS, reactive oxygen species; SCFAs, short-chain fatty acids; SOD, superoxide dismutase; SOD1, superoxide dismutase 1; SYN, synaptophysin; TGF-β, transforming growth factor beta; Th17, T helper 17 cell; TJP, tight junction protein; TLR4, Toll-like receptor 4; TNF-α, tumor necrosis factor alpha; Treg, regulatory T cell; Wnt, Wingless-related integration site; ZO-1, zonula occludens 1.
